# Evaluating the role of alpha cell dysregulation in the progression to type 2 diabetes using mathematical simulations

**DOI:** 10.1007/s00125-025-06524-1

**Published:** 2025-09-09

**Authors:** Vijaya Subramanian, Arthur S. Sherman, Jens J. Holst, Filip K. Knop, Tina Vilsbøll, Jonatan I. Bagger

**Affiliations:** 1 Institute for Computational Medicine, Johns Hopkins University, Baltimore, MD USA; 2 Laboratory of Biological Modeling, National Institute of Diabetes, Digestive and Kidney Diseases, Bethesda, MD USA; 3 Novo Nordisk Foundation Center for Basic Metabolic Research, Faculty of Health and Medical Sciences, University of Copenhagen, Copenhagen, Denmark; 4 Department of Biomedical Sciences, Faculty of Health and Medical Sciences, University of Copenhagen, Copenhagen, Denmark; 5 Center for Clinical Metabolic Research, Gentofte Hospital, University of Copenhagen, Hellerup, Denmark; 6 Department of Clinical Medicine, Faculty of Health and Medical Sciences, University of Copenhagen, Copenhagen, Denmark; 7 Novo Nordisk, Bagsværd, Denmark; 8 Clinical Research, Steno Diabetes Center Copenhagen, Herlev, Denmark

**Keywords:** 2 h plasma glucose, Alpha cell dysregulation, Beta cell compensation, Beta cell failure, Fasting glucose, Glucagon, HbA_1c_, Incretins, Insulin resistance, Longitudinal progression

## Abstract

**Aims/hypothesis:**

Alpha cell dysregulation is an integral part of type 2 diabetes pathophysiology, increasing fasting as well as postprandial glucose concentrations. Alpha cell dysregulation occurs in tandem with the development of insulin resistance and changes in beta cell function. Our aim was to investigate, using mathematical modelling, the role of alpha cell dysregulation in beta cell compensatory insulin secretion and subsequent failure in the progression from normoglycaemia to type 2 diabetes defined by ADA criteria.

**Methods:**

We developed a physiological model of glucose homeostasis, whereby the fast dynamics of glucose, insulin and glucagon are coupled to the dynamics of beta cell functional mass (a product of individual beta cell functional capacity and mass). Beta cell functional mass exhibits an initial compensatory increase in response to hyperglycaemia, followed by an eventual decline due to glucotoxicity. Alpha cell dysregulation, defined as increased glucagon secretion and lowered glucagon suppression resulting in hyperglycaemia, was introduced to varying extents, and simulations were carried out to assess the effects on beta cell functional mass over a 20 year period.

**Results:**

The simulations were carried out under conditions of moderate, mild or no alpha cell dysregulation. The parameters representing insulin resistance, glucagon secretion and suppression for an individual with normoglycaemia obtained from previously published work were evolved over a period of 20 years to the mean values observed in type 2 diabetes. The model was validated by visually matching the beta cell functional mass obtained from the simulations of the disease progression model to previously published parameters. Those parameters were obtained from fits of a model of OGTTs to data from a cross-sectional cohort that spanned the spectrum from normoglycaemia to type 2 diabetes. We found that mild alpha cell dysregulation elicited robust beta cell compensation, resulting in controlled postprandial glucose excursions despite the development of insulin resistance. Moderate alpha cell dysregulation initially enhanced compensation but eventually accelerated the progression to type 2 diabetes. Alpha cell dysregulation impacted the time course of the standard markers of diabetes (fasting glucose, 2 h plasma glucose and HbA_1c_) during disease progression.

**Conclusions/interpretation:**

The early stages of alpha cell dysregulation led to robust beta cell functional mass compensation driven by elevated fasting glucose. When the dysregulation progressed further, glucose levels rose to levels of glucotoxicity, exacerbating beta cell functional mass loss and accelerating the onset of type 2 diabetes. The various markers of diabetes (fasting glucose, 2 h plasma glucose and HbA_1c_) differed in terms of the prediction of timing of onset of disease, depending on the extent of alpha cell dysregulation.

**Graphical Abstract:**

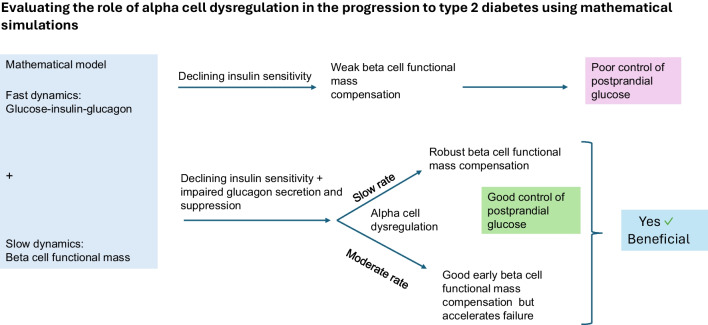

**Supplementary Information:**

The online version contains peer-reviewed but unedited supplementary material available at 10.1007/s00125-025-06524-1.


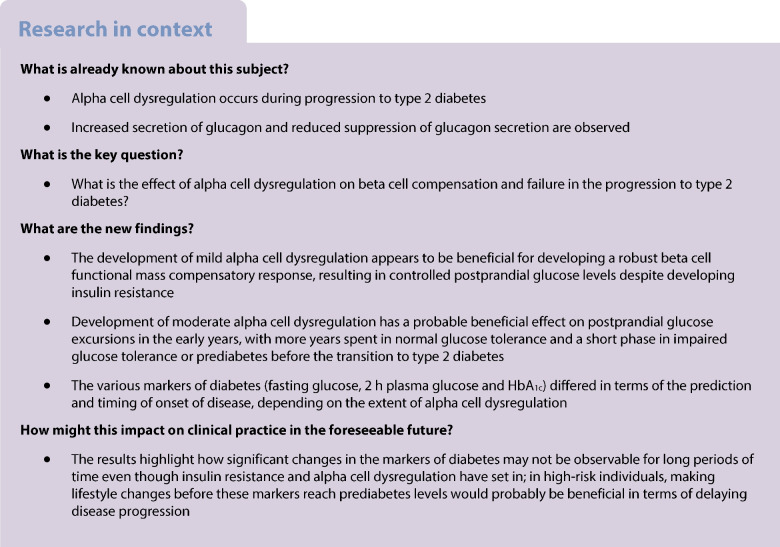


## Introduction

Progression from normoglycaemia to type 2 diabetes is characterised by a confluence of events. The trajectory has been shown to be associated with genetic predisposition, prior gestational diabetes in women, overweight and obesity, visceral fat depots and ectopic fat deposition [1–5].

One of the physiological consequences of overweight and obesity is the development of peripheral insulin resistance [4, 6–8]. Development of insulin resistance is associated with increased insulin secretion [9] due to increased beta cell functional mass (compensation), which maintains normal glucose levels but is typically followed by a decrease in beta cell functional mass (failure of compensation), accompanied by increasing plasma glucose levels, and, eventually, overt type 2 diabetes [10]. Beta cell functional mass represents the product of total beta cell mass and individual beta cell functional capacity. In addition to insulin resistance and beta cell dysfunction and mass loss, both elevated secretion and inadequate suppression of glucagon secretion from alpha cells in response to glucose occur. This hyperglucagonaemia leads to elevated fasting and postprandial glucose levels, thus contributing to the development of type 2 diabetes [11–14]. An important unanswered question is the role of this alpha cell dysregulation in beta cell functional mass compensation and subsequent failure [15, 16].

Several mathematical models [17–21] have been used to describe the progression to type 2 diabetes. In the first such model, developed by Topp et al [17], the glucose dynamics controlled by insulin was coupled to the slow dynamics of beta cell mass. Beta cell mass was assumed to be determined by a fine balance between beta cell proliferation and death, which are both glucose dependent, and compensation and failure in beta cell mass were demonstrated to occur with the development of insulin resistance. Although this seminal paper [17] provided the concept used in subsequent models of what might drive beta cell mass loss and transition to type 2 diabetes, it had shortcomings in terms of parameter selection, the form of the compensation in the slow dynamics, the exaggerated expansion of beta cell mass, and the timescale of progression to type 2 diabetes. The role of hepatic glucose production in type 2 diabetes was not investigated sufficiently, nor was the effect of meals on disease progression. Subsequent papers [18–21] tackled some of these issues, but none of the previous studies addressed the role of alpha cell dysregulation in the development of type 2 diabetes or included incretins in the fast dynamics.

Here we aimed to study the roles of alpha cell dysregulation and incretins through their effect on beta cell functional mass compensation and failure in the development of type 2 diabetes. Crucially, we use a validated model of glucose, insulin and glucagon dynamics that includes incretins to describe the fast dynamics [22, 23]. The parameters in the model were estimated by fitting data from a cross-sectional cohort of BMI-matched (± 1 kg) individuals of European descent ranging from normoglycaemia (age 57 years [38–74]; BMI 29 kg/m^2^ [26–33]; fasting plasma glucose 5.3 mmol/l [4.8–5.7]; HbA_1c_ 5.4% [5.0–5.7]) to type 2 diabetes (age 57 years [40–75]; BMI 29 kg/m^2^ [25–34]; fasting plasma glucose 7.7 mmol/l [7.0–8.9]; HbA_1c_ 7.0% [6.2–8.4]; duration of diabetes 8 months [6–36], based on WHO criteria; means and range) [23–25]. These parameters provided the information needed to study the effect of impairment in glucagon secretion and suppression and the incretin effect in the transition from normoglycaemia to type 2 diabetes. We introduce a new improved description of beta cell functional mass dynamics that depends on changes in both glucose-stimulated insulin secretion (GSIS) and incretin-potentiated insulin secretion (IPIS), and parameterise based on the cross-sectional data. We also used a realistic timescale of disease progression that covers a span of 10–20 years [26], depending on the insult to the system.

## Methods

### Mathematical model and parameters

#### Model

The equations describing glucose homeostasis comprise two parts: one describing the fast dynamics of glucose, insulin and glucagon under the perturbations of daily meal ingestion which we based on a previously published model, and one for the dynamics of beta cell functional mass. We included the incretin glucose-dependent insulinotropic polypeptide (GIP) to describe postprandial dynamics. In our previous papers [22, 23], we found that unique parameters related to both incretins could not be determined simultaneously, thus we use the parameters for GIP only, as it has been shown to be the stronger potentiator of insulin secretion under postprandial conditions, and is also the likely cause for the diminished incretin effect in type 2 diabetes [27, 28]. Longitudinal changes are assessed by varying parameters related to insulin sensitivity and glucagon secretion and suppression (representative of alpha cell dysregulation), and observing the response in beta cell functional mass. In previous models, the only challenge to the beta cells was reduced insulin sensitivity.

Equations 1–3, describing the fast dynamics, are discussed in detail in the paper by Subramanian et al [23]. Briefly, Eq. 1 represents glucose dynamics as a sum of four terms. *G*, *I* and *A* represent glucose, insulin and glucagon concentrations, respectively. The first two terms describe glucose- and insulin-dependent glucose disposal, respectively. *S*_G_ is the glucose-dependent glucose removal rate constant. The parameter *a*_1_ is a measure of insulin sensitivity. The third term represents glucagon-dependent hepatic glucose production, where *a*_2_ is a measure of glucagon action in the liver. The last term, *R*_*a*_[*t*, *p*]/*V*, represents influx of glucose from the gut (described in electronic supplementary material [ESM] Methods).

Equation 2 describes insulin dynamics. The first term is the rate of insulin degradation where *n*_1_ is the degradation rate constant. The second term represents GSIS. The dependence of GSIS on glucose is given by ψ in Eq. 4. The last term represents IPIS. As IPIS is known to occur in a glucose dependent manner, it is represented by a product of glucose and incretin concentrations. The rate constants *Beta*_γ1_ and *Beta*_γ3GIP_ are measures of beta cell functional mass associated with GSIS and IPIS, respectively. In this model, the functional mass is a composite term accounting for the role of both mass and function in insulin secretion, as there are no data available from humans that help to discriminate between the two.

Equation 3 describes glucagon dynamics. The first term represents the rate of glucagon degradation where *n*_2_ is the degradation rate constant. The second term represents glucose-dependent glucagon secretion. We used a simplified version of glucagon secretion given by φ in Eq. 4. Other models of glucagon secretion consider paracrine modulation of glucagon secretion by insulin [29, 30]. As the relative contribution of intrinsic relative to paracrine regulation of glucagon secretion is suggested to be higher under high glucose [31], we did not consider paracrine control, which likely plays a modulatory role, in this paper.

1 $$\frac{\text{d}G[t]}{\text{d}t}=-\left({S}_{G}+{a}_{1}I\left[t\right]\right)G\left[t\right]+{a}_{2}A\left[t\right]+{R}_{a}[t,p]/V$$

2 $$\frac{\text{d}I[t]}{\text{d}t}=-{n}_{1}I\left[t\right]+{Beta}_{{\upgamma }_{1}}\uppsi \left(G\left[t\right]\right)+ \text{ } {Beta}_{{\upgamma }_{3\text{GIP}}}GIP\left[t\right]G[t]$$

3 $$\frac{\mathrm dA\lbrack t\rbrack}{\mathrm dt}=-n_2A\left[t\right]\;+\;\gamma_2\mathrm\varphi\!\left(G\lbrack t-\mathrm\tau{}_1\rbrack\right)$$

4 $$\mathrm{where}\;\mathrm\psi\!\left(G\lbrack t\rbrack\right)=1.5\;{G\left(t\right)}^{1.3}/\left(17^{1.3}+{G\left(t\right)}^{1.3}\right)\;\mathrm{and}\;\mathrm\varphi\!\left(G\left[t-{\mathrm\tau}_1\right]\right)=\mathrm e^{-k_1G\;\left(t-{\mathrm\tau}_1\right)}$$

5 $$\frac{{\text{d}Beta}_{x}}{\text{d}t}=({c}_{1}\text{SND}\left[\mu ,\sigma ,\alpha ,G[t]\right]{-{c}_{2})Beta}_{x}\left(UB-{Beta}_{x}\right)/UB$$

6 $$\text{SND}\left(\mu ,\sigma ,\alpha ,G\right)=\frac{2}{\sigma \sqrt{2\uppi }}{\text{e}}^{-\frac{{(t-\mu )}^{2}}{2{\upsigma }^{2}}}{\int }_{-\infty }^{\alpha \left(\frac{G-\mu }{\sigma }\right)}\frac{1}{\sqrt{2\uppi }}{\text{e}}^{\frac{-{z}^{2}}{2}}\text{d}z$$

Equation 5 describes the slow dynamics of beta cell functional mass in response to changing glucose levels. GSIS and IPIS, while sharing a common initiating step, follow different amplifying pathways and are treated as distinct. The two terms, *Beta*_γ1_ and *Beta*_γ3GIP_, in Eq. 2 are thus expected to respond differentially to the changing glucose levels that occur in response to the developing insulin resistance or alpha cell dysregulation. The rates of change of these two parameters are assumed to follow similar dynamics, with *x* in Eq. 5 representing either γ_1_ or γ_3GIP_. The term within the first parentheses in the equation represents the compensation factor and determines the growth and decline of beta cell functional mass in response to changing glucose levels. An upper bound (*UB*) was introduced in Eq. 5 to prevent unbounded growth of functional mass.

**Fig. 1:**
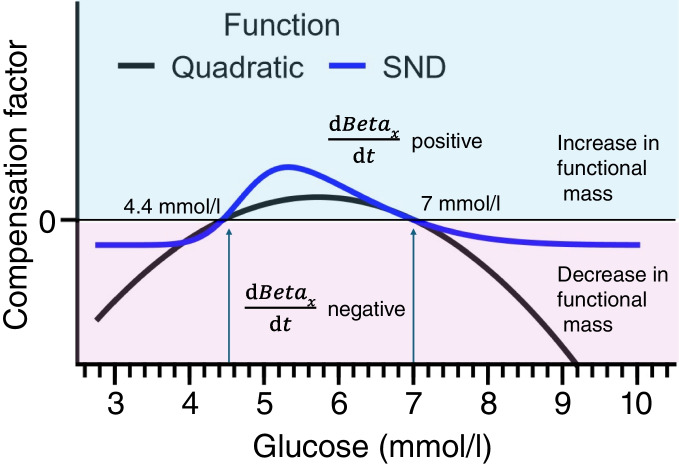
Plot of the compensation factor (arbitrary units) in Eq. 5 vs plasma glucose. The blue curve depicts the SND and the black curve represents the parabola in the Topp model [17]. The derivative of beta cell functional mass is positive in the blue region and negative in the pink region

In the Topp model [17], the compensation factor in Eq. 5, which is considered to represent beta cell mass (not functional mass), is given by a quadratic function of glucose. The form of the function is based on the postulate that beta cell mass is driven by a balance between beta cell proliferation and death rates and depicted by the black curve in Fig. 1. Between the two steady states of the beta equation, the beta cell proliferation rate exceeds the death rate (blue region in Fig. 1). When glucose levels exceed the upper steady state, the death rate exceeds the proliferation rate, leading to a decline in beta cell mass (pink region in Fig. 1). However, the predicted decline in beta cell mass is very steep in response to glucose levels above the upper steady state, leading to an implausibly rapid loss of beta cell mass once the threshold is crossed. Additionally, there is no upper bound to the compensation, so beta cell mass increases in an unrealistic manner when the decline in insulin sensitivity is slow. However, during the progression to type 2 diabetes, both beta cell mass and function are observed to change [9, 16, 32, 33]. In our model, we assume that compensation is driven by a composite term, beta cell functional mass (*Beta*_*x*_), as it is uncertain as to how much, mass or function, independently contributes to disease progression [9, 16, 32, 33], and we use a similar but improved formalism to represent it. We use the skew normal distribution (SND, Eq. 6) [34], a function that stays bounded at all glucose values, as the compensation factor, to moderate beta cell functional mass dynamics. The shape of the function (Fig. 1, Eq. 6) may be adjusted using the parameters μ (mean), *σ* (width) and α (skewness), making it highly versatile and amenable to describing different compensatory behaviours. In addition, once the upper steady state is crossed, the decline in functional mass is more modest, because the tail of the SND flattens out, in line with the observed preservation of some beta cell function as disease progresses [9, 33]. The constant *c*_1_ scales the magnitude of the response, and *c*_2_ is a shift that is used to set the normal steady state and threshold level for transition to type 2 diabetes.

#### Parameter selection

**Fig. 2:**
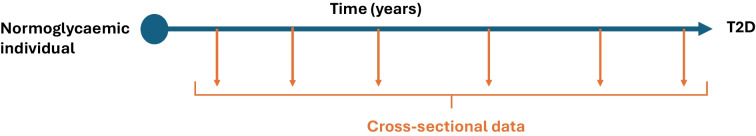
A depiction of how cross-sectional data may represent longitudinal progression to disease from normoglycaemia to type 2 diabetes (T2D). The orange arrows represent individuals in the cross-sectional data who are at different stages of progression to disease. If the aetiology of disease is similar in all members of the cohort, these arrows can represent different time points in the longitudinal progression

The parameters used in the fast dynamics equations (Eqs. 1–4) for the longitudinal model simulations were chosen based on previously published fits [23] to OGTTs in a cross-sectional cohort [24] (see representative examples in ESM Fig. 1). Figure 2 shows how cross-sectional data may be used to set the parameters governing longitudinal progression to disease. If we assume that the disease aetiology is similar among the individuals within the cohort ranging from those with normoglycaemia to those with type 2 diabetes, each may be thought of as representing a different time point (orange arrows) in the longitudinal progression (blue line) to disease of an individual with normoglycaemia. The variation in the parameters representing insulin sensitivity (*a*_1_ in Eq. 1), glucagon suppression (*k*_1_ in Eq. 4), glucagon secretion (γ_2_ in Eq. 3) and insulin secretion (*Beta*_*x*_ in Eq. 2) in these different individuals (equivalent to different time points) determines how an individual with normoglycaemia may progress to type 2 diabetes. The starting values of the parameters in the simulations were chosen from an individual with normoglycaemia and the highest insulin sensitivity and lowest fasting glucose (FG) within the cohort. The parameters *a*_1_, γ_2_ and/or *k*_1_ were then evolved, based on Eqs. 7–9, to the mean values observed in type 2 diabetes (ESM Tables 1 and 2) to observe the effect of the various dysregulations on beta cell functional mass dynamics.

The cross-sectional data were also used to parameterise the compensation factors and upper bound in the slow dynamics (Eq. 5). The upper bounds of *Beta*_*x*_ were set to three times the upper limit observed in the cohort studied as there is evidence in the literature of higher insulin values (and correspondingly higher *Beta*_*x*_) in certain groups, e.g. Southwest Native Americans [35], during the compensatory phase. As the individuals evolved from normoglycaemia to type 2 diabetes, the parameters in the compensation factor, namely *μ*, *σ*, *α*, *c*_1_ and *c*_2_, were chosen by trial and error to reflect the compensatory insulin secretion observed in the cross-sectional cohort. This method had to be adopted because cross-sectional data do not have the requisite temporal information for regression. First the steady-state values of 4.4 and 7 mmol/l (Fig. 1) were approximately set by adjusting the width σ and shift *c*_2_. The lower stable steady state was set at the lower end of the normal baseline glucose range of 4-6 mmol/l and the upper unstable steady state at 7 mmol/l, the value at which individuals transition to type 2 diabetes based on ADA criteria. Then all parameters, including *c*_1_, were tuned such that the individual with normoglycaemia transitions through the cross-sectional states observed in the cohort to type 2 diabetes. This was done by comparing simulation and experimental plots of *Beta*_*x*_ vs insulin sensitivity (*a*_1_) as shown in the Results. An example is given in ESM Fig. 1, which shows three individuals from the cohort representing normal, intermediate and type 2 diabetes parameter values (given in ESM Table 3). As described in Subramanian et al [23], some participants in the cohort with normoglycaemia had lower insulin sensitivity and showed compensatory insulin secretion. During the simulations, an individual with normoglycaemia similar to control participant 5 (CP 5, see ESM Fig. 1) transitions through compensatory insulin levels similar to those of control participant 4 (CP 4) to mean values of insulin secretion as in type 2 diabetes participant 1 (T2D 1), which are determined by the functional mass compensation *Beta*_*x*_.

7 $$\frac{\text{d}{a}_{1}}{\text{d}t}=-c\;{a}_{1}$$

8 $$\frac{\text{d}{k}_{1}}{\text{d}t}=-{c}_{supp}\;{k}_{1}$$

9 $$\frac{\text{d}{\upgamma }_{2}}{\text{d}t}={c}_{sec}\;{\upgamma }_{2}$$

#### Longitudinal simulations

The simulations were carried out using a carbohydrate influx of 225 g per day. This was divided equally between breakfast, lunch and dinner at 06:00 hours, 12:00 hours and 18:00 hours. Varying levels of dysregulation were introduced through Eqs. 7–9, and simulations were carried out for periods of 20–35 years. The short time delay, τ_1_, of the order of minutes in Eq. 4 was not found to have an effect on disease progression over a time span of years and was not included in the simulations presented. The simulations were performed using Mathematica version 14.2 [36].

#### Clinical trial registration

The original RCT, of which this is a secondary analysis, is registered at www.ClinicalTrials.gov as NCT00529048. The clinical trial was conducted by JIB, TV, FKK and JJH co-authors of this paper. This secondary analysis is original work that is different from all previous analyses of the data from this RCT.

## Results

**Fig. 3:**
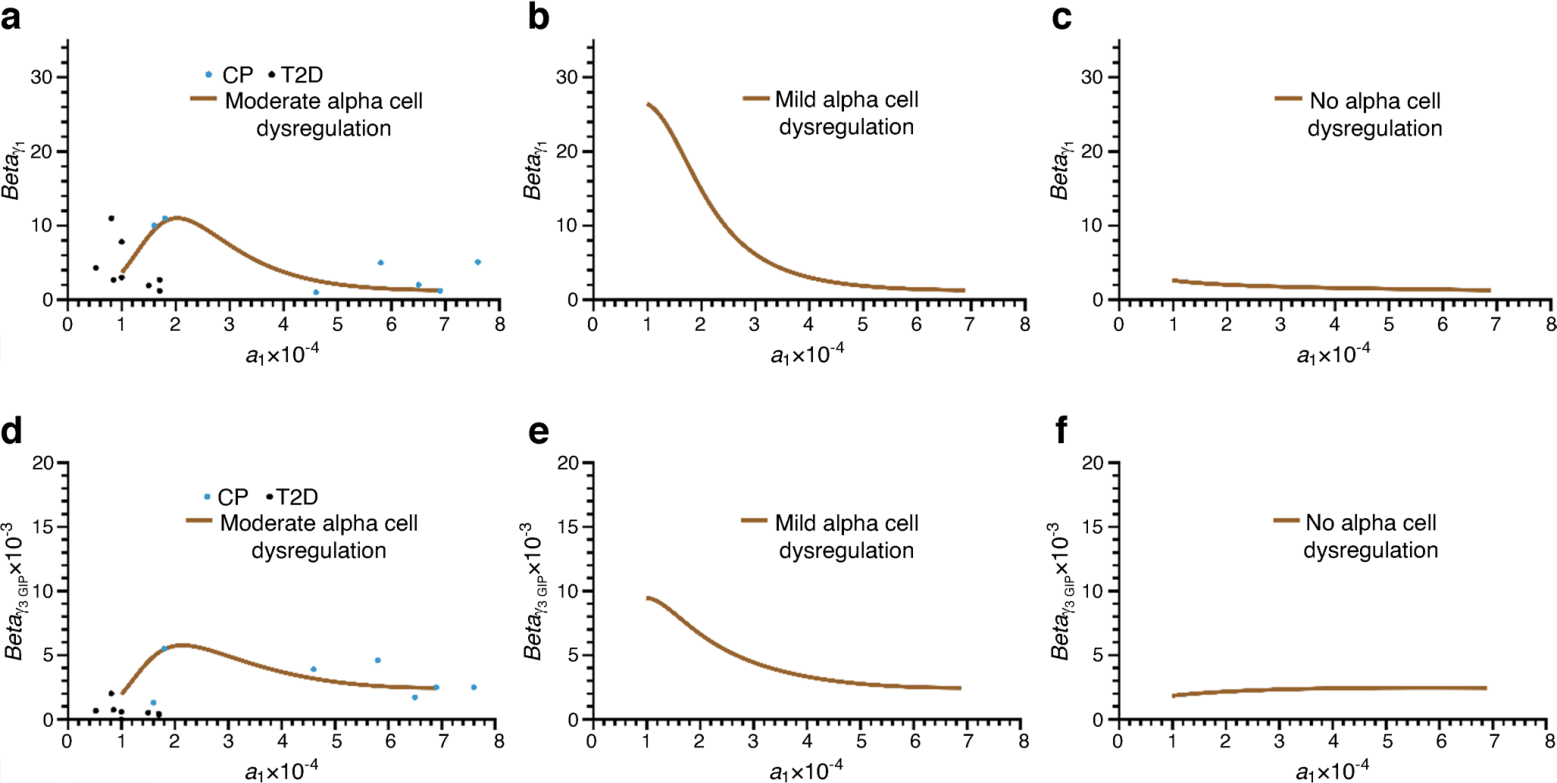
Plots of beta cell functional mass parameters *Beta*_γ1_ (**a**–**c**) and *Beta*_γ3GIP_ (**d**–**f**) vs the insulin sensitivity factor *a*_1_. In (**a**) and (**d**), the same parameters determined from the fit to data from the cross-sectional cohort (blue dots, control participants [CP]; black dots, type 2 diabetes [T2D]) are plotted to show the goodness of fit with the simulation-derived parameters when alpha cell dysregulation evolves at a moderate rate in addition to declining insulin sensitivity. When mild alpha cell dysregulation is introduced, stronger compensation is observed (**b**, **e**). When there is no alpha cell dysregulation, there is no significant compensation (**c**, **f**)

**Fig. 4:**
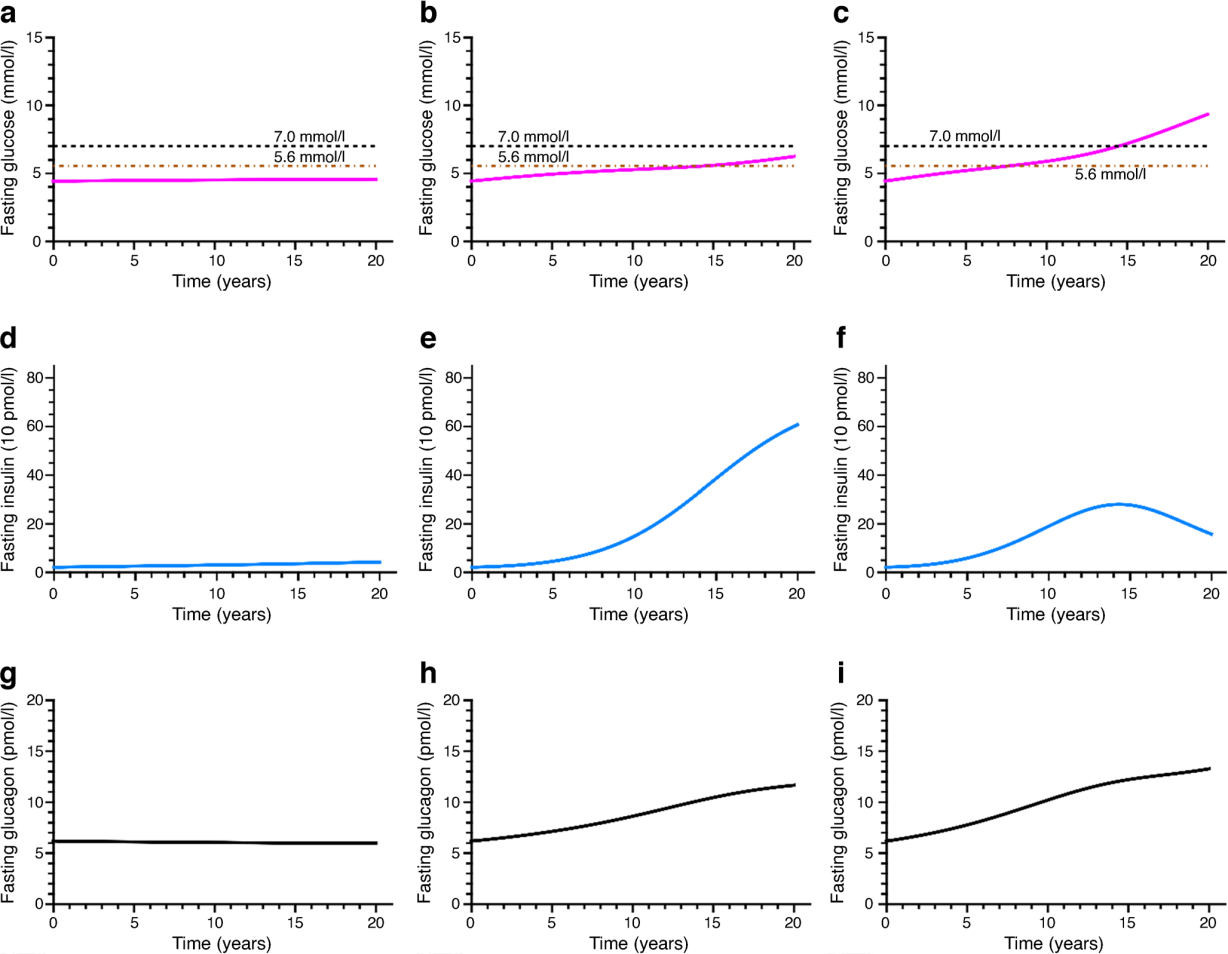
Plots of FG, fasting insulin and fasting glucagon vs time during the longitudinal simulations. (**a**, **d**, **g**) No alpha cell dysregulation; (**b**, **e**, **h**) mild alpha cell dysregulation; (**c**, **f**, **i**) moderate alpha cell dysregulation. Insulin sensitivity declines at the same rate in all three scenarios

**Fig. 5:**
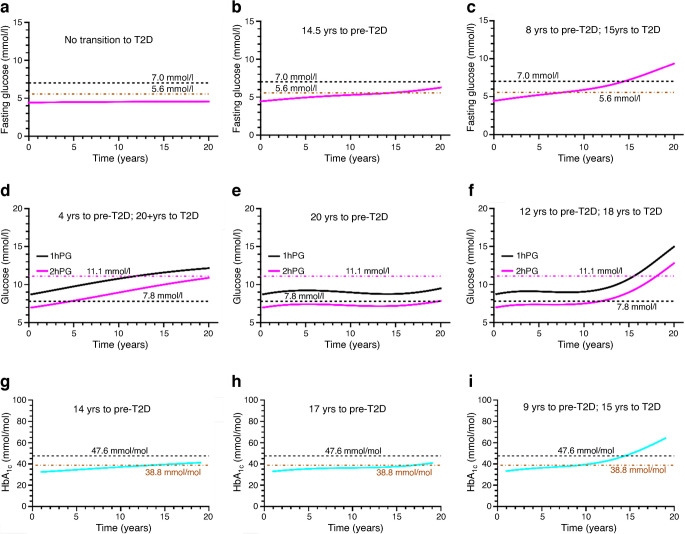
Plots of FG, plasma glucose (1hPG and 2hPG) and HbA_1c_ vs time as obtained from the longitudinal simulations. (**a**, **d**, **g**) No alpha cell dysregulation; (**b**, **e**, **h**) mild alpha cell dysregulation; (**c**, **f**, **i**) moderate alpha cell dysregulation. Insulin sensitivity declines at the same rate in all three scenarios. T2D, type 2 diabetes; yrs, years

**Fig. 6:**
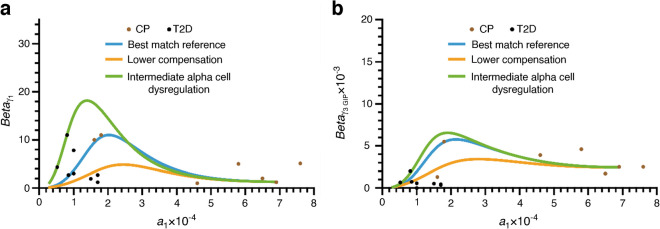
Plots of *Beta*_γ1_ and *Beta*_γ3GIP_ vs *a*_1_ for the three cases: blue curve, moderate alpha cell dysregulation; green curve, intermediate alpha cell dysregulation; orange curve, lower beta cell compensation. The variance in the parameters from the cross-sectional cohort (brown dots [CP] and black dots [T2D]) is explained by the differences in the extent of alpha cell dysregulation (green curve) and compensatory capacity (orange curve) relative to the reference (blue curve). CP, control participants; T2D, type 2 diabetes

**Fig. 7:**
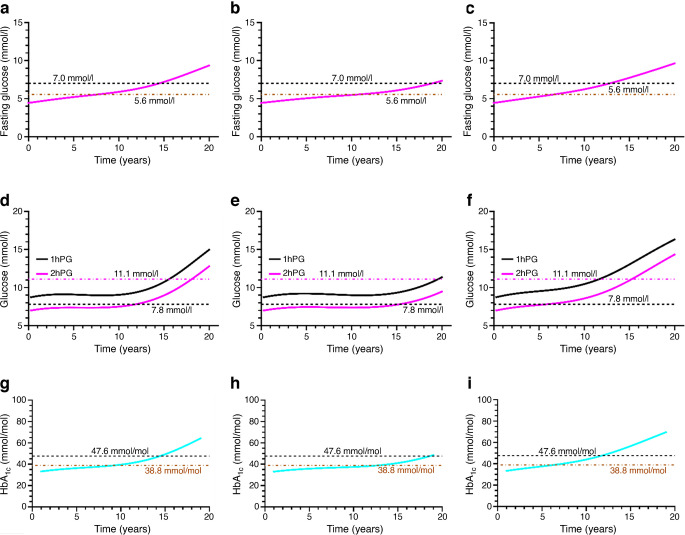
Plots of FG, plasma glucose (1hPG and 2hPG) and HbA_1c_ as a function of time for three scenarios: moderate alpha cell dysregulation in the reference best match (**a**, **d**, **g**), intermediate alpha cell dysregulation (**b**, **e**, **h**) and lower compensatory capacity (**c**, **f**, **i**). Relative to the reference best match in (**a**), (**d**) and (**g**), the transition to type 2 diabetes via all three metrics is slower when the compensation is higher (intermediate alpha cell dysregulation, same compensatory capacity) and faster when the compensation is lower (lower compensatory capacity, similar alpha cell dysregulation)

In the first set of simulations (Figs 3, 4 and 5), the role of alpha cell dysregulation, in addition to the development of insulin resistance, in the progression to type 2 diabetes is investigated. In the second set of simulations (Figs 6 and 7), the effects on disease progression of the rate of decline in alpha cell function as well as a lower capacity for beta cell functional mass compensation, in addition to insulin sensitivity decline, are evaluated.

### Alpha cell dysregulation

The glucose, insulin and glucagon dynamics over a period of 2 days at the start of the simulation are shown in ESM Fig. 2. The influx of glucose from the gut was assumed to follow the profile of the glucose influx in an OGTT, as this would represent an upper bound of glucose arrival from the gut after a meal. Correlations between plasma glucose values obtained from mixed-meal tolerance tests and OGTT have been found to be very high [37–39]. The incretin secretory profile used to model IPIS was also based on the response observed in the OGTT. The starting values of all parameters, including those in the glucose influx term, were based on previously published work [23] and are presented in ESM Table 4; fixed parameters are indicated as such.

A series of simulations were carried out for a period of 21 years for three scenarios: declining insulin sensitivity (*a*_1_) evolving combined with (path 1) development of moderate alpha cell dysregulation (increasing magnitude of glucagon secretion combined with declining suppression by glucose, with both γ_2_ and *k*_1_ evolving), corresponding to the behaviour observed in the cross-sectional cohort studied; (path 2) mild alpha cell dysregulation (increasing magnitude of glucagon secretion, with γ_2_ evolving and *k*_1_ kept constant); and (path 3) no alpha cell dysregulation (*k*_1_ and γ_2_ constant). The parameters of the normoglycaemic individual chosen at the start of the simulation were allowed to evolve to the mean values observed in type 2 diabetes (ESM Table 2) at rates determined by *c*, *c*_supp_ and *c*_sec_ in Eqs. 7–9 (ESM Table 5).

The response of the beta cell functional mass to changes in the three cases above occurs via two terms, *Beta*_γ1_ and *Beta*_γ3GIP_, which represent GSIS and IPIS. The differential equations, Eqs. 5, describing these two terms have the same form, but the parameters used are different (ESM Tables 5 and 6). The scaling factor and shift in both equations were adjusted such that the individual with normoglycaemia transitions through the compensatory levels of insulin secretion via GSIS and IPIS observed in the cross-sectional cohort along path 1 (see above) to type 2 diabetes. Path 1 follows a hypothetical individual from normoglycaemia to type 2 diabetes as described in the Methods. In Fig. 3(a, d), the continuous curve represents the values of *Beta*_γ1_ and *Beta*_γ3GIP_ plotted against insulin sensitivity (*a*_1_), all obtained from solving the coupled Eqs. 1–9 of the longitudinal model following path 1. Moving from right to left, as insulin sensitivity declines and moderate alpha cell dysregulation develops over a period of 20 years, beta cell functional mass increases for a few years until a maximum is reached and then declines as compensation fails. This is the point where the transition to type 2 diabetes occurs. The rising arm of the curve corresponds to the blue region in Fig. 1 between 4.4 and 7 mmol/l where the compensation factor is positive. The turning point and transition to type 2 diabetes occur when the compensation factor becomes negative at 7 mmol/l. In Fig. 3(a, d), the values of *Beta*_γ1_ and *Beta*_γ3GIP_ obtained by fitting the fast dynamics (Eqs. 1–3) to the OGTT data from the cross-sectional reference cohort (blue dots, normoglycaemic; black dots, type 2 diabetes) are also plotted against insulin sensitivity (*a*_1_). There is good visual concordance between the longitudinal simulation and the fits of the cross-sectional data.

When alpha cell dysregulation is mild (path 2 above), both the GSIS and IPIS components of functional mass compensation increase significantly as insulin sensitivity declines, and reach a maximum towards the end of the simulation (Fig. 3b, e). When there is no alpha cell dysregulation (path 3), there is no appreciable compensation in beta cell functional mass (Fig. 3c, f).

In Fig. 4, the FG, insulin and glucagon values are presented as functions of time for the three cases of alpha cell dysregulation presented in Fig. 3. When there is progressive loss of insulin sensitivity but no alpha cell dysregulation, FG values do not rise (Fig. 4a). When there is mild alpha cell dysregulation in addition to insulin sensitivity decline, FG levels rise slowly over 20 years (Fig. 4b). When there is moderate alpha cell dysregulation, FG levels rise slowly for a few years, then rise sharply to exceed the diabetes threshold of 7 mmol/l (Fig. 4c).

Figure 4d–f shows fasting insulin profiles over the simulation period. In the absence of alpha cell dysregulation, fasting insulin does not change much (Fig. 4d). With mild alpha cell dysregulation, insulin increases robustly, then levels off (Fig. 4e). When alpha cell dysregulation is moderate, fasting insulin rises slowly, reaches a maximum and then declines (Fig. 4f). Fasting glucagon levels (Fig. 4g–i) reflect the degree of alpha cell dysregulation. When there is no dysregulation, glucagon stays close to baseline (Fig. 4g), increasing when dysregulation is mild (Fig. 4h) or moderate (Fig. 4i).

Figure 5 shows FG, 1 h and 2 h postprandial glucose (1hPG and 2hPG) and HbA_1c_ for the same simulations as in Fig. 4. HbA_1c_ was calculated from the average glucose values over a 3 month period [40] at the end of each simulation year. The times to development of prediabetes and type 2 diabetes under various degrees of alpha cell dysregulation are also shown. Figure 5a shows that transition to pre-type 2 diabetes or type 2 diabetes defined by FG does not occur when there is no alpha cell dysregulation. When alpha cell dysregulation is mild (Fig. 5b), the transition to pre-type 2 diabetes takes approximately 15 years, and there is no transition to type 2 diabetes even after 21 years. When alpha cell dysregulation is moderate (Fig. 5c), transition to pre-type 2 diabetes takes 8 years and transition to type 2 diabetes takes 15 years (based on FG).

Figure 5d–f shows longitudinal progression of 2hPG. When there is no alpha cell dysregulation, transition to pre-type 2 diabetes takes 4 years and transition to type 2 diabetes takes over 20 years (Fig. 5d). With mild alpha cell dysregulation, transition to pre-type 2 diabetes is significantly delayed, taking 20 years, and there is no transition to type 2 diabetes (Fig. 5e). With moderate alpha cell dysregulation, the transition to prediabetes takes 12 years and transition to type 2 diabetes takes 18 years (Fig. 5f).

Figure 5g–i show the longitudinal progression of HbA_1c_. The transition to pre-type 2 diabetes takes 14 years with no alpha cell dysregulation (Fig. 5g), 17 years with mild alpha cell dysregulation (Fig. 5h) and 9 years with moderate alpha cell dysregulation (Fig. 5i). Only with moderate dysregulation does transition to type 2 diabetes based on HbA_1c_ occur (after 15 years).

Table 1 shows that FG, insulin, glucagon, 2hPG and HbA_1c_ from the simulation with moderate alpha cell dysregulation after 20 years fall within the range of the experimentally determined mean values in type 2 diabetes individuals in the cross-sectional cohort.

**Table 1:** Simulated and experimental FG, insulin and glucagon concentrations, and 2hPG and HbA_1c_ values

Parameter	Simulation value	Experimental value
FG (mmol/l)	9.4	7.9 (7.2–8.9)
FG (mg/dl)	170	143 (130–160)
Fasting insulin (pmol/l)	140	94 (48–176)
Fasting glucagon (pmol/l)	13	13 (7–24)
2hPG (mmol/l)	13	16 (12–21)
2hPG (mg/dl)	235	288 (208–381)
HbA_1c_ (mmol/mol)	64	42 (44–68)
HbA_1c_ (%)	8.0	7.0 (6.2–8.4)

Values are those obtained from the simulation with moderate alpha cell dysregulation after 20 years, and mean experimental values with range for individuals with type 2 diabetes from the cross-sectional cohort

### Effects of rate of decline of alpha cell dysregulation and compensation capacity

We have shown that the model fits well the degree of compensation observed across the spectrum of the cross-sectional cohort. In Fig. 6(a, b), we show that some of the outlier points corresponding to higher or lower degrees of compensation can also be captured if some parameters are varied. The blue curve represents moderate alpha cell dysregulation as shown in Fig. 3 and Table 1, and represents the best visual match. The orange curve shows a lower degree of compensation, achieved by adjusting the parameters *c*_1_ and *c*_2_ in Eq. 5. The green curve shows a higher level of compensation, achieved with intermediate alpha cell dysregulation relative to the reference (achieved by reducing the parameter *c*_sec_ in Eq. 9). The parameter values are given in ESM Table 5.

The effects of the changes in Fig. 6 on the markers of diabetes are shown in Fig. 7. The transition to type 2 diabetes occurs more slowly when the compensation is higher (intermediate alpha cell dysregulation; Fig. 7b, e, h) and more quickly when the compensation is lower (c_1_ and c_2_ adjusted; Fig. 7c, f, i) relative to the best match of the cohort (Fig. 7a, d, g). When alpha cell dysregulation develops more slowly (intermediate alpha cell dysregulation), the time spent in normal glucose tolerance (NGT) is greater.

## Discussion

Progression from NGT to diabetes has long been associated with the development of insulin resistance and a concomitant compensatory response and subsequent failure of beta cell functional mass. Here we have investigated the roles of various players, especially alpha cell dysregulation and incretin-potentiated compensation and failure, in the progression to type 2 diabetes. We also investigated the effect of the various dysregulations on FG, 1hPG and 2hPG and HbA_1c_, as there has been a lot of discussion on how these measures of disease state align with each other temporally and which may be an earlier predictor of disease.

While simulations under fasting-only conditions may be instructive, as in previous studies [17, 20, 41], a realistic longitudinal simulation must include meals. In addition to the effect that meals may have on compensation, their inclusion is the only way to assess changes in glucose tolerance over time and to address the role of incretins. We have presented two sets of simulations that include meals. In the first set of simulations, we established the role of alpha cell dysregulation in disease progression. In the second set, we explained the variance in the cross-sectional data based on the degree of alpha cell dysregulation and the magnitude of compensation capacity. We show that the parameters related to beta cell functional mass in a cross-sectional cohort drawn from a population of European descent in Denmark, who were BMI-matched and ranged from normoglycaemic to type 2 diabetes, could be effectively described by the longitudinal model developed here. With inclusion of the development of moderate alpha cell dysregulation, in addition to declining insulin sensitivity, a hypothetical normoglycaemic individual was shown to transition to type 2 diabetes, while passing through the stages of functional mass compensation and decline in line with parameters from that cross-sectional cohort. The beta cell functional mass changes observed in the cross-sectional cohort could not be matched when alpha cell dysregulation was not included in the model. Both GSIS and IPIS went through a compensatory phase, with subsequent failure, in the longitudinal progression to type 2 diabetes, in line with the cohort parameters. We also showed that, with inclusion of moderate alpha cell dysregulation, fasting and postprandial glucose, as well as insulin and glucagon levels, fall within the range observed in the cross-sectional cohort (Table 1).

Experimentally, varying levels of glucagon secretion and suppression have been observed across cohorts of varying glycaemic status [14, 42, 43] including ours [44]. We investigated the effects of changes in the magnitude of alpha cell dysregulation on disease progression by varying the rates of decline of glucagon suppression and secretion. With no alpha cell dysregulation, the hypothetical normoglycaemic individual transitions to type 2 diabetes via 2hPG levels after a long phase in pre-type 2 diabetes, because the compensatory response in this case is minimal, leading to an increase in postprandial glucose levels and eventual disease. In contrast, we showed that, when mild alpha cell dysregulation develops over time, there is a very robust regulatory response in terms of beta cell functional mass because of the modest elevation in FG levels. Thus, the hypothetical normoglycaemic individual barely transitioned to pre-type 2 diabetes based on 2hPG values over the 20 year time frame, and more time was spent in NGT, which is of great benefit clinically. This individual did not transition to type 2 diabetes based on FG or HbA_1c_ levels over the same time frame. Moderate alpha cell dysregulation, on the other hand, exacerbated pathophysiology, leading to a more rapid transition to type 2 diabetes. The advantage over the case with no alpha cell dysregulation is that the number of years spent in NGT is longer, with a short phase in pre-type 2 diabetes before transition to type 2 diabetes. We posit that mild and moderate alpha cell dysregulation in the early years following development of insulin resistance leads to a strong compensatory response from beta cell functional mass, and is beneficial in controlling postprandial glucose levels. These patterns of changes in FG, 2hPG and HbA_1c_ have been observed in two longitudinal studies [45, 46]. These observations have potentially important implications for prevention of type 2 diabetes.

We found that the various markers of diabetes, FG, 2hPG and HbA_1c_, differed in terms of prediction and timing of onset of disease, depending on the primary mechanism leading to disease. When both insulin resistance and alpha cell dysregulation occurred in parallel, as in the cross-sectional cohort studied, 2hPG lagged behind FG and HbA_1c_ in predicting the development of type 2 diabetes. Similarly, when there was mild alpha cell dysregulation, FG was the earlier predictor. With both mild and moderate alpha cell dysregulation, the time spent in NGT was longer before the transition to pre-type 2 diabetes. When there was no alpha cell dysregulation, 2hPG was an earlier predictor than FG or HbA_1c_. The time spent in NGT was much shorter before the transition to pre-type 2 diabetes. Thus, the differences in degree of alpha cell dysregulation were a major determinant of the timing of higher FG, 2hPG and HbA_1c_ levels.

Although a role for hyperglucagonaemia has long been recognised [12, 47–49], this is the first mechanistic, longitudinal model of progression that addresses the role of glucagon. This is an advance over previous longitudinal models in terms of the careful parametrisation used here based on our validated model of the fast dynamics as well as inclusion of alpha cell dysregulation and incretins. This model also represents an advance over the phenomenological hyperbolic relationship between measures of beta cell function and insulin sensitivity that has been used to show differences across cross-sectional cohorts [50, 51]. In the previous study [51], empirical hyperbolic curves were overlaid over cross-sectional data, and individuals were hypothesised to move along or between curves in the transition to type 2 diabetes. Our physiological model produces trajectories in the insulin sensitivity/beta cell functional mass compensation space that provide a direct explanation for the various pathways to diabetes based on the dysregulations that can occur.

Use of cross-sectional data to model longitudinal progression has been applied in other areas of biology [52]. Here we showed that cross-sectional data can be used as a surrogate for longitudinal data in modelling type 2 diabetes disease progression. The ready availability of cross-sectional data from various cohorts could be used to ascertain the various modalities of progression to type 2 diabetes: for example, in lean, overweight and obese individuals, in different sexes or ethnic groups, or for post-gestational diabetes. Future possibilities could also involve testing the model on longitudinal data [35]. The current model does not distinguish between hepatic and peripheral insulin sensitivity, include paracrine control of glucagon secretion [29–31], or include changes in *S*_G_ (glucose-dependent glucose disposal) [53] or hepatic insulin clearance [54], which have been implicated in the progression to type 2 diabetes; their inclusion may be an option for improving the model in the future.

In the second set of simulations, we showed that the model could readily account for lower and higher degrees of compensation relative to the average behaviour observed in the cross-sectional Danish cohort (Fig. 6). We showed how individuals starting at the same level of insulin secretion and insulin sensitivity transition from NGT to type 2 diabetes along different dysregulation pathways. These cases resemble the differences seen in different global populations: lower compensation is seen in East Asians compared with Europeans [55], and higher compensation than in Europeans is seen in South Asians and people of African descent, including African Americans [56–58]. This finding suggests that the model has sufficient flexibility and explanatory power to address the full range of pathways to type 2 diabetes seen in different populations and could aid in optimising therapy for groups and individuals.

## Supplementary Information

Below is the link to the electronic supplementary material.

ESM (PDF 487 KB)

## Data Availability

Data will be made available by the corresponding authors upon reasonable request.

## Abbreviations

1hPG: 1 h plasma glucose
2hPG: 2 h plasma glucose
FG: Fasting glucose
GIP: Glucose-dependent insulinotropic polypeptide
GSIS: Glucose-stimulated insulin secretion
IPIS: Incretin-potentiated insulin secretion
NGT: Normal glucose tolerance
SND: Skew normal distribution

## Code availability

Computer code used for the simulations will be posted in the Johns Hopkins Research Data Repository: 10.7281/T19XBTVF.

## Authors’ relationships and activities

ASS has a collaboration with Eli Lilly Inc. to use mathematical modelling. JIB has served on scientific advisory panels and/or received research support from Novo Nordisk and Zealand Pharma. JJH is a consultant with Novo Nordisk, and is also on advisory boards for Novo Nordisk. He is the founder and an advisory board member for Antag Therapeutics. FKK has served on scientific advisory panels and/or been part of speakers’ bureaus for, served as a consultant to, holds stocks in, and/or has received research support from 89bio, Amgen, Altimmune, AstraZeneca, Bayer, Boehringer Ingelheim, Carmot Therapeutics, Chr. Hansen, Cytoki Pharma, Eli Lilly, Gubra, MedImmune, MSD/Merck, Mundipharma, Norgine, Novo Nordisk, MedImmune, Pharmacosmos, Sanofi, Structure Therapeutics, Zealand Pharma and Zucara, is a co-founder of and minority shareholder in Antag Therapeutics, and is currently employed full-time by Novo Nordisk. TV has served on scientific advisory panels, been part of speakers’ bureaus, served as a consultant to and/or received research support from Amgen, AstraZeneca, Boehringer Ingelheim, Eli Lilly, Gilead, GSK, MSD/Merck, Novo Nordisk, Roche, Sanofi, Sun Pharmaceuticals and Zealand Pharma. VS declares that there are no relationships or activities that might bias, or be perceived to bias, their work.
