# Supplementary material for: Evaluating the role of alpha cell dysregulation in the progression to type 2 diabetes using mathematical simulations
Source: Diabetologia. 2025 Sep 9;68(11):2595–608. doi: 10.1007/s00125-025-06524-1 (PMC12534288; doi:10.1007/s00125-025-06524-1)
Supplement: Supplementary file 1 — ESM (PDF 487 KB) [file 125_2025_6524_MOESM1_ESM.pdf]

Electronic Supplementary Material

ESM Methods

Description of the glucose arrival from the gut [1]: A skew normal distribution was used to model $R_a[t,p]$ with parameters $p \rightarrow \{\mu, \sigma, \alpha\}$:

$$SND(\mu,\sigma,\alpha,t) = \frac{2}{\sigma\sqrt{2\pi}} e^{-\frac{(t-\mu)^2}{2\sigma^2}} \int_{-\infty}^{\alpha\left(\frac{t-\mu}{\sigma}\right)} \frac{1}{\sqrt{2\pi}} e^{\frac{-x^2}{2}} dx \quad (1)$$

Depending on whether the glucose profile post OGTT had a single peak or two peaks, one or two SND's were used to fit the data.

$$R_a[t,p] = b_1 SND(\mu_1,\sigma_1,\alpha_1,t) + b_2 SND(\mu_2,\sigma_2,\alpha_2,t) \quad (2)$$

The integral of the glucose arrival rate is constrained to be equal to a fraction of the ingested glucose as follows:

$$\int_0^T R_a\,[t,p]dt = \frac{D \times f}{BW} \quad (3)$$

D is the ingested glucose dose in mg, f is the fraction of the dose that is assumed to be absorbed and available after transiting the liver[2] and BW is the body weight in kg. V in Eqn. (1) is the volume of distribution per unit body weight (dl/kg). V was set to 1.35, D 75000 mg, and f to .86. If the simulation is done in mmol/l, then divide Ra[t,p] by 18 to convert to mmol/l.

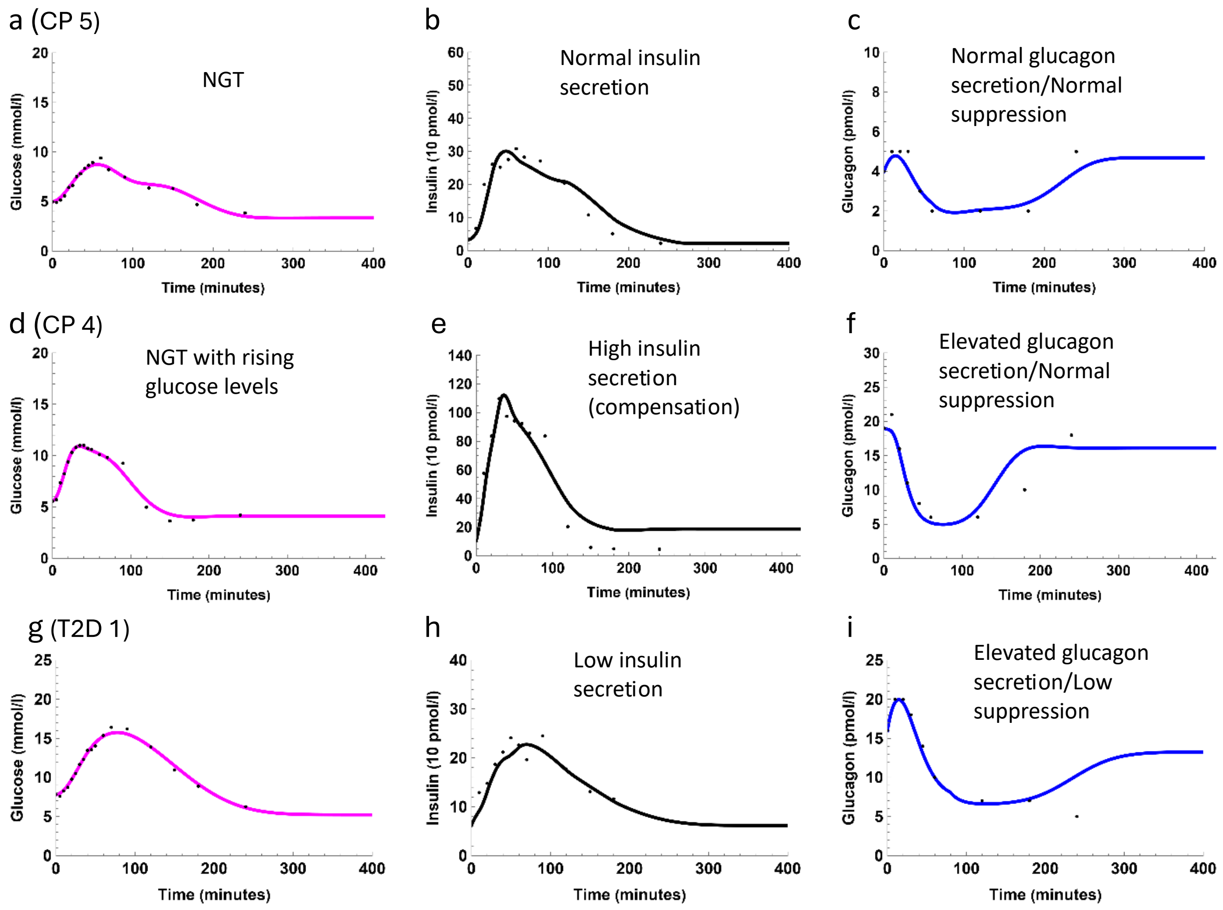

ESM Figure 1: The glucose, insulin and glucagon profiles of a normoglycaemic individual (panels a-c), an individual with borderline normoglycaemia showing significant compensatory insulin secretion (panels d-f) and an individual with T2D (panels g-i).[1]

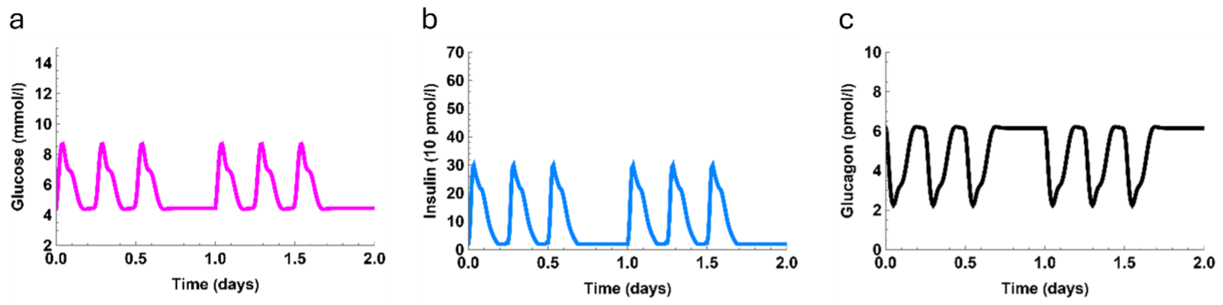

ESM Figure 2: Dynamics of glucose, panel a, insulin, panel b, and glucagon, panel c subject to three 75g carbohydrate meals a day with a rest period in-between at the beginning of the longitudinal simulation.

Controls		
Parameter	Mean (SEM)	Range
α_1 ($\times 10^{-4}$) (10 pmol/l min)$^{-1}$	5.0 (.92)	1.6-7.6
α_2 mmol/l (pmol/l min)$^{-1}$	0.01 (.0016)	.004-.017
k_1 (mmol/l)$^{-1}$	.30 (.076)	.07-.72
γ_1 10 pmol/l min^{-1}	5.0 (1.5)	1.0-11.
γ_2 pmol/l min^{-1}	2.8 (.64)	1.5-6.2
γ_{3GIP} (mmol/l min)$^{-1}$	.056 (.01)	.023-.18

ESM Table 1: Mean values of the parameters in the control group (normoglycaemic) of the cross-sectional cohort where a_1 is a measure of insulin sensitivity, a_2 glucagon action at the liver, k_1 glucagon suppression constant, γ_1, glucose dependent insulin secretion beta-cell functional mass constant, γ_2 glucagon secretion constant, γ_{3GIP} incretin potentiated insulin secretion constant.[1]

T2D		
Parameter	Mean (SEM)	Range
a_1(×10^{-4}) (10 pmol/l min)$^{-1}$	1.1 (.16)	0.52-1.7
a_2 mmol/l (pmol/l min)$^{-1}$	0.014 (0.0035)	0.0034-0.03
k_1 (mmol/l)$^{-1}$	0.15 (0.01)	0.12-0.20
γ_1 10 pmol/l min^{-1}	4.3 (1.2)	3.0-11.
γ_2 pmol/l min^{-1}	5.2 (0.74)	1.2-13.
γ_{3GIP} (mmol/l min)$^{-1}$	0.0099 (0.0038)	0.0-.025

ESM Table 2: Mean values of the parameters in the T2D group of the cross-sectional cohort where a_1 is a measure of insulin sensitivity, a_2 glucagon action at the liver, k_1 glucagon suppression constant, γ_1 glucose dependent insulin secretion beta-cell functional mass constant, γ_2 glucagon secretion constant, γ_{3GIP} incretin potentiated insulin secretion constant.[1]

Subject #	Glucagon suppression k_1 (mmol/l)$^{-1}$	Insulin sensitivity α_1 (10 pmol/l min)$^{-1}$	GSIS γ_1 (10 pmol/l min^{-1})	Glucagon secretion γ_2 (pmol/l min^{-1})	IPIS γ_{3GIP} ((mmol/l min)$^{-1}$)	Glucagon action α_2 (mmol/l (pmol/l min)$^{-1}$)
CP 5	0.25	6.9×10^{-4}	1.7	1.5	4.5×10^{-2}	0.014
CP 4	0.24	1.8×10^{-4}	11.	5.8	9.9×10^{-2}	0.004
T2D 1	0.14	1.0×10^{-4}	3.0	5.7	11.0×10^{-3}	0.005

	Fasting glucose mmol/l (mg/dl)	2hr-plasma glucose mmol/l (mg/dl)	HbA_{1c}
CP 5	5.1 (92)	6.4 (115)	35.5 mmol/mol<br>5.4 %
CP 4	5.7 (103)	5.0 (90)	36.6 mmol/mol<br>5.5 %
T2D 1	7.7 (139)	13.9 (250)	55.2 mmol/mol<br>7.2 %

ESM Table 3: Parameter values in three individuals CP 5 (normoglycaemic), CP 4 (borderline normoglycaemic) and T2D 1 (Type 2 diabetes) from modeling the cross-sectional cohort[1]. **HbA_{1c}, Fasting Glucose (FG) and 2hr Plasma glucose (2hr-PG) values of the same individuals. CP-control participant. T2D-type 2 diabetes.**[1]

Parameter	Initial Value
a_1 (10 pmol/l min)$^{-1}$	0.00069
a_2 mmol/l (pmol/l min)$^{-1}$ (Fixed)	0.01
$Beta_{\gamma 1}$ (10 pmol/l min^{-1})	1.2
γ_2 (pmol/l min^{-1})	1.5
$Beta_{\gamma 3GIP}$ (mmol/l min)$^{-1}$	0.043
S_G (min^{-1}) (Fixed)	0.014
n_1 (min^{-1}) (Fixed)	0.14
n_2 (min^{-1}) (Fixed)	0.08
BW (kg) (Fixed)	85.0
k_1 (mmol/l)$^{-1}$	0.25
b_1 min^{-1} (Fixed)	5.3
b_2 min^{-1} (Fixed)	3.0
μ_1 min (Fixed)	20..0
μ_2 min (Fixed)	100.
σ_1 min (Fixed)	40.0
σ_2 min (Fixed)	45.0
α_1 dimensionless (Fixed)	2.1
α_2 dimensionless (Fixed)	1.7
$UB\gamma_1$ (10 pmol/l min^{-1}) (Fixed)	35.0
$UB\gamma_{3GIP}$ (mmol/l min)$^{-1}$ (Fixed)	0.31

ESM Table 4: The initial values of the parameters in Eqns. 1-3 and 5 used in the simulations.

Parameter	Simulation				
	Moderate alpha cell dysfunction	Intermediate alpha cell dysfunction	Mild alpha cell dysfunction	No alpha cell dysfunction	Lower Compensation
c_1 (SF $Beta_{\gamma 1}$) * min^{-1}	0.61	0.61	0.61	0.61	0.46
c_2 (shift $Beta_{\gamma 1}$)** min^{-1}	0.47	0.47	0.47	0.47	0.35
c_1 (SF $Beta_{\gamma 3GIP}$)* min^{-1}	0.54	0.54	0.54	0.54	0.35
c_2 (shift $Beta_{\gamma 3GIP}$)** min^{-1}	0.50	0.50	0.50	0.50	0.35
c (day^{-1})	0.00025	0.00025	0.00025	0.00025	.00025
c_{supp} (day^{-1})	0.000067	0.000067	0.000067	0.0	0.000067
c_{sec} (day^{-1})	0.00015	0.0001	0.0	0.0	0.00015

ESM Table 5: Parameters c_1 and c_2 in the beta-cell compensation factors in Eqs. 5 and the constants c, c_{supp} and c_{sec} which define the rate at which insulin sensitivity, glucagon suppression and secretion declines in Eqs. 7-9 used in the different simulations are presented in columns 2-6.

(*×.0009; **×10^{-6})

simulation	μ mmol/l (mg/dl)	σ mmol/l (mg/dl)	α (dimensionless)
Moderate, mild, intermediate no alpha-cell dysfunction, $Beta_{\gamma 1}$	4.6 (84.0)	1.5 (27.0)	3.5
Moderate, mild, intermediate, no alpha-cell dysfunction, $Beta_{\gamma 3GIP}$	4.5 (82.0)	1.9 (33.7)	3.5
Lower compensation, $Beta_{\gamma 1}$	4.6 (84.0)	1.5 (27.0)	3.5
Lower compensation, $Beta_{\gamma 3GIP}$	4.5 (82.0)	1.8 (32.5)	3.5

ESM Table 6: The values of μ, σ, and α for the SNDs in Eq. 5 in the different simulations are given in the table. The values for the compensation factor in the $Beta_{\gamma 1}$ and $Beta_{\gamma 3GIP}$ equations are presented separately.

[1] Subramanian V, Bagger JI, Harihar V, et al. An extended minimal model of OGTT: estimation of α- and β-cell dysfunction, insulin resistance, and the incretin effect. Am J Physiol Endocrinol Metab 2024;326:E182–205. https://doi.org/10.1152/ajpendo.00278.2023.

[2] Caumo A, Bergman RN, Cobelli C. Insulin sensitivity from meal tolerance tests in normal subjects: A minimal model index. Journal of Clinical Endocrinology and Metabolism 2000; 85:4396–402. https://doi.org/10.1210/jcem.85.11.6982.
